# Perceptions on Oral Ulcers From Facebook Page Categories: Observational Study

**DOI:** 10.2196/45281

**Published:** 2023-05-08

**Authors:** Suguna Simhadri, Sriha Yalamanchi, Sean Stone, Mythily Srinivasan

**Affiliations:** 1 Department of Oral Pathology, Medicine and Radiology Indiana University Purdue University at Indianapolis Indianapolis, IN United States; 2 Indiana University School of Dentistry Indiana University Purdue University at Indianapolis Indianapolis, IN United States

**Keywords:** oral ulcer, internet, Facebook, information, apthous stomatitis, cold sore

## Abstract

**Background:**

Oral ulcers are a common condition affecting a considerable proportion of the population, and they are often associated with trauma and stress. They are very painful, and interfere with eating. As they are usually considered an annoyance, people may turn to social media for potential management options. Facebook is one of the most commonly accessed social media platforms and is the primary source of news information, including health information, for a significant percentage of American adults. Given the increasing importance of social media as a source of health information, potential remedies, and prevention strategies, it is essential to understand the type and quality of information available on Facebook regarding oral ulcers.

**Objective:**

The goal of our study was to evaluate information on recurrent oral ulcers that can be accessed via the most popular social media network—Facebook.

**Methods:**

We performed a keyword search of Facebook pages on 2 consecutive days in March 2022, using duplicate, newly created accounts, and then anonymized all posts. The collected pages were filtered, using predefined criteria to include only English-language pages wherein oral ulcer information was posted by the general public and to exclude pages created by professional dentists, associated professionals, organizations, and academic researchers. The selected pages were then screened for page origin and Facebook categories.

**Results:**

Our initial keyword search yielded 517 pages; interestingly however, only 112 (22%) of pages had information relevant to oral ulcers, and 405 (78%) had irrelevant information, with ulcers being mentioned in relation to other parts of the human body. Excluding professional pages and pages without relevant posts resulted in 30 pages, of which 9 (30%) were categorized as “health/beauty” pages or as “product/service” pages, 3 (10%) were categorized as “medical & health” pages, and 5 (17%) were categorized as “community” pages. Majority of the pages (22/30, 73%) originated from 6 countries; most originated from the United States (7 pages), followed by India (6 pages). There was little information on oral ulcer prevention, long-term treatment, and complications.

**Conclusions:**

Facebook, in oral ulcer information dissemination, appears to be primarily used as an adjunct to business enterprises for marketing or for enhancing access to a product. Consequently, it was unsurprising that there was little information on oral ulcer prevention, long-term treatment, and complications. Although we made efforts to identify and select Facebook pages related to oral ulcers, we did not manually verify the authenticity or accuracy of the pages included in our analysis, potentially limiting the reliability of our findings or resulting in bias toward specific products or services. Although this work forms something of a pilot project, we plan to expand the project to encompass text mining for content analysis and include multiple social media platforms in the future.

## Introduction

Mouth ulcers are painful sores that affect any portion of the mouth, and more than 1 ulcer can occur at the same time. They are commonly due to local factors, such as trauma resulting from cheek biting, dental appliance use, and stress. They are often managed at home without professional care, although they can also occur as a result of mucocutaneous diseases, oral precancer, and cancer [1]. Oral ulcers cause discomfort when eating, swallowing, and speaking, affecting the smooth functioning of everyday life. Yet, since recurring oral ulcers are usually self-healing, people commonly tend to put up with them or refer to alternate treatments rather than seek professional advice [2,3]. In these times of easy internet access, solutions, potential remedies, and prevention strategies are often sought from social media networks [4,5]. However, since the content published in social media is not regulated or peer reviewed, it may not be accurate or reliable [6].

The purpose of Facebook is to empower people to share and make society a more increasingly networked place. Currently, more than 1 billion people are registered Facebook users, with more than 175 million adult users in the United States, and Facebook is broadly popular among all demographic groups [7]. It has been reported that over 96% of Facebook users access the site via mobile devices; as such, information is literally in the palms of their hands [8]. Although Facebook is primarily used to stay up to date with news and current events, seeking and sharing health information are common uses of the Facebook channel among the public [9].

Little is known about the broader representation of health conditions on Facebook. In a comprehensive study, Farmer et al [10] searched all Facebook groups, using a list of search terms comprising 11 of the most prevalent noncommunicable diseases identified by the World Health Organization, and found that respiratory, diabetes, cardiovascular disease, and digestive disease groups consisted of the highest number of groups, while groups related to malignant neoplasms had the most members. De la Torre-Díez et al [11] searched for groups related to 3 diseases with highest public burden (breast cancer, colorectal cancer, and diabetes) and found that “prevention” groups that seek to raise awareness of or money for a disease were the most popular groups for all 3 diseases.

Concerns about the quality of social media information and its impact on individual health outcomes have been raised by both health professionals and the public. It has been suggested that the anonymity of sources, sponsors, financial interests, the absence of medical credentials and reputable research, the use of testimonials as evidence, and outdated or incomplete information should raise suspicion about the reliability of information [12]. For instance, a patient at a dental office who presented with an ulcer on the cheek shared her internet experience. She said that she searched for possible pain-killing treatments for the ulcer on the internet. Her friend had suggested a Facebook page, and while browsing pages related to mouth ulcers on Facebook, she discovered quite a few pages that were advertising products for curing oral ulcers. In contrast, there were few other pages that discussed home cures for treating ulcers, but she was not sure which ones were appropriate. The goal of our study was to evaluate information on recurrent oral ulcers that can be accessed via the most popular social media network—Facebook.

## Methods

### Facebook Accounts

We created 2 Facebook accounts. The first account was used to conduct the specific search for this study, and the second account was used for reference, that is, to cross-check the information retrieved by the first account. We performed a cross-sectional study in 3 stages. In the first phase, we conducted a search to determine the number of pages related to oral ulcers on Facebook.

### Search Strategy

A Facebook page is a collection of posts that are created by a person or an organization with the intention to share innovative ideas about a particular topic on a regular basis. If the person creating the posts has a public account, the posts are normally accessible to everyone; otherwise, they appear only on the timelines of individuals who follow that person's private account. We began our search by collecting posts from pages in Facebook that used the keywords. We then analyzed the information by applying the inclusion and exclusion criteria. To avoid search bias while collecting pages and posts, 2 anonymous accounts were created. Two trained evaluators conducted the searching and selection of websites from March 28 to March 29, 2022; both evaluators were dentists. A systematic search of posts on the Facebook social media platform was undertaken, using the search terms “Stomatitis Aphthous,” “Aphthous Stomatitides,” “Aphthous Stomatitis,” “Stomatitides Aphthous,” “Ulcer Aphthous,” “Aphthous Ulcer,” “Aphthous Ulcers,” “Ulcers Aphthous,” “Aphthae,” “Canker Sore,” “Canker Sores,” “Sore Canker,” “Sores Canker,” “Periadenitis Mucosa Necrotica Recurrens,” “Oral ulcer and mouth ulcer,” and “Oral ulcer or mouth ulcer.” No restriction was placed on the locations of postings, and the origins of the postings were not always evident. After the keywords were used to search Facebook, relevant pages were saved as PDF files within 24 hours to avoid any changes. All of the posts on the pages that were part of this study were gathered.

### Data Collection

The initial search retrieved non–dental-related and dental-related pages. We then applied predefined eligibility criteria to include only English-language pages that were posted by individuals affected by oral ulcers or support groups. Pages created by professionals, professional organizations, and academic researchers and pages with information on or images of nonoral and nondental ulcers were excluded (Textbox 1). The selected pages were then screened for page origin and Facebook categories.

**The eligibility criteria used to determine relevant data for page selection.**

**Criteria for authors**

- Inclusion
  Public authors affected by oral ulcers or those supporting others with oral ulcers
- Exclusion
  Absence of oral or dental symptoms or images
  Pages authored by dental health care professionals or academic researchers
  Professional organizations

**Criteria for language, type of post, and audience**

- Inclusion
  English language
  Written word
  Friends and family, general public, and health care professionals
- Exclusion
  Not in English language
  Photographs and emojis

### Statistical Analysis

The data collected were presented as percentages of the distribution of selected Facebook pages with respect to page origin and Facebook categories.

### Ethical Considerations

Ethics approval was not applicable, as no personal identifiable data were accessed or analyzed.

## Results

### Text Mining

**Figure 1:**
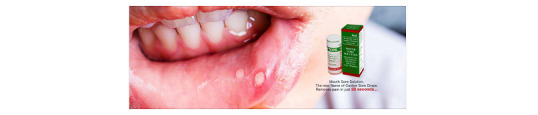
One of the Facebook pages retrieved via the search strategy and used in this pilot study.

**Figure 2:**
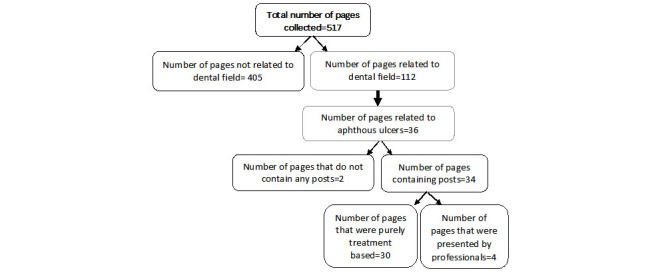
A flowchart of the selection process. The flowchart shows the total number of pages collected via keyword searching, the number of dental-related pages, the number of non–dental-related pages, the number of pages related to aphthous ulcers, the number of pages that did not contain posts, the number of pages that were purely treatment based, and the number of pages presented by professionals.

All posts were anonymized to maintain confidentiality and prevent bias during the analysis. Following familiarization with the data set, manual text mining was performed to allow for the selection of pages related to oral ulcers and the exclusion of pages related to oral manifestations of oral precancer, cancer, infectious diseases, and mucocutaneous lesions. A keyword search yielded a total of 517 pages that included 112 pages on oral ulcers. Following the application of the eligibility criteria, 70 posts were excluded, including posts from dental professionals and 9 educational posts. Further, 405 of 512 pages were excluded because the pages were not related to dentistry, with 78% (405/517) of these pages providing information related to widespread ulcers in the body, and the rest of the excluded pages were in languages other than English. After text mining, a total of 32 dental pages on oral ulcers were identified. These 32 dental-related pages were screened, and 2 pages were excluded due to a lack of any posts. Of note, 26 pages included posts on therapies for oral ulcers, and 4 were pages created by dental professionals (Figures 1 and 2).

### Facebook Categories and Page Origin

In Facebook page creation, choosing the right category is considered very important, as the main category appears next to the identity of the page owner (business, organization, or individual), making it easy to connect this information [13]. Out of 30 Facebook dental pages and categories, we found that 30% (9/30) of the sites in our data set were categorized as “health/beauty” pages or as “Dentist & Dental Office Beauty, Cosmetic & Personal Care” pages, 10% (3/30) were categorized as “medical & health” pages, and an approximate of 17% (5/30) were categorized as “community” pages. This suggests that Facebook, in oral ulcer information dissemination, has been primarily used as an adjunct to business enterprises for marketing or for enhancing access to a product (Textbox 2). Although we could not find the sources for 3 of the posts in our data set, it was interesting to note that all pages categorized as “community” pages originated from the United States, while “product” category pages originated from England, Philippines, Australia, and India (3 pages each) as well as from South Africa, France, and Uganda (1 page each).

**The Facebook dental pages and their categories.**

**Page names and Facebook categories**

1. A Spoonful of Oral Medicine (category: Medical & Health – Education)
2. Acioclair Ulcer Treatment (category: Product/service)
3. Can this canker sore get more fans than Florida Atty. General Bill McCollum (category: Community)
4. Canker Mouth Sores Solution – Pasig (category: Product/service)
5. Canker Shield (category: Product/service)
6. Canker Sore Cures (category: Community)
7. Canker Sore Management (category: Community)
8. Canker Sore Support (category: Community)
9. Canker Sore Treatment (category: Health/beauty)
10. Canker Sores Begone Stick (category: Health/beauty)
11. Canker Sores Treatments, Preventions, and Cures (category: Book)
12. Canker Sore treatment (category: Health/beauty)
13. Debacterol Canker Sore Treatment (category: App page)
14. Dissa-Peer Tablets and Cream – Nutritional Supplements for Mouth Ulcers (category: Company)
15. Dr. Harshil Shah-Root canal treatment expert and Implantologist (category: General Dentist – Teeth Whitening Service)
16. Filmstead – Mouth Ulcer Patch (category: Medical Service – Pharmacy/Drugstore – Family Doctor)
17. Finally, a cure for Mouth Ulcers (category: Medical & Health – Community)
18. How to Get Rid of a Canker Sore (category: Medical Company)
19. How To Get Rid Of Canker Sores (category: Health/beauty)
20. JVR Mouth Sore Solution (category: Pharmaceuticals)
21. Alternative & Holistic Health Service (category: Alternative & Holistic Health Service – Medical Supply Store)
22. Mouth ulcer problem 1 solution (category: Health/beauty)
23. Mouth Ulcers Shop (category: Dentist & Dental Office Beauty, Cosmetic & Personal Care)
24. Not Having Canker Sores (category: Organization)
25. Oral Care – The Laser Way (category: Medical & Health)
26. Oral Pathology & Medicine (category: Education website)
27. Oralieve (category: Health & wellness website – Medical & Health)
28. Sabka dentist (category: Health/beauty – Health & wellness website)
29. Sodium Lauryl Sulfate dries your skin and gives mouth ulcers (category: Health/beauty)
30. The Canker Spanker (category: Health/beauty)

## Discussion

### Principal Findings

Oral ulcers are usually painful and uncomfortable and, therefore, make it challenging to eat, drink, and speak. In addition, they can also be unsightly, resulting in embarrassment or self-consciousness [2,3]. A sizable majority of adult internet users report looking for health information on the internet. Facebook has been on the top of the social network sites to be accessed by most people over the past 3 years and is the most accessed social media site for news among American adults [7,14]. Our analysis was restricted to the information available on mouth ulcers (ie, recurrent oral ulcers) in Facebook pages. The level of participation by people with a post indicates how much they are interested in a topic, as well as how much influence the topic has on society. 70% (21/30) pages were found under the keyword “sore, canker,” whereas there were no pages for the keyword “Periadenitis Mucosa Necrotica Recurrens.” Notably, there was little information on the prevention of oral ulcers, long-term treatment, and complications of oral ulcers. Furthermore, the Facebook channel was used most often as an adjunct business tool for products and services, particularly by account holders outside of the United States. Interestingly, in contrast, most pages (24/80, 30%) originating from the United States (not included in this study) were discussion blogs on oral cancer.

Categories in Facebook are used to grow a business, brand, or organization. Hence, it is intriguing to observe that 40% (12/30) of the pages chose the “health/beauty” category or the “product/service” category, suggesting that the primary use of Facebook was mostly for aesthetics and potential remedies. Further, this may indicate that oral ulcers are not perceived as significantly serious enough to search for related information on other professional websites, such as the WebMD website.

### Limitations

Social network channels, including Facebook, are dynamic and change continuously. Our study was restricted to posts created within a specified time and had the following limitations. First, only posts were taken into consideration; we did not consider private messages, the number of likes, and private groups. Second, our study does not provide information about the extent to which people were benefiting from the information presented on Facebook. Although we made efforts to identify and select Facebook pages related to oral ulcers, we did not manually verify the authenticity or accuracy of the pages included in our analysis, potentially limiting the reliability of our findings and resulting in bias toward specific products or services. Finally, it is likely that an in-depth content analysis via text mining could be more useful for assessing the potential of Facebook pages as objective information sources on oral ulcers [15]. Although this work forms something of a pilot project, we hope to expand the project to encompass text mining for content analysis and include multiple social media platforms in the future.

### Conclusions

Observations from our qualitative study showed that the Facebook platform has been used partly as a marketing tool for the sharing of information on oral ulcers by unofficial pages and by business or quasi-business entities. Future quantitative and analytic studies are needed to elucidate the complete usefulness of this social network channel.

## Data Availability

Data sharing is not applicable to this article, as no data sets were generated or analyzed during this study.
